# Polyaniline-Supported Nickel Oxide Flower for Efficient Nitrite Electrochemical Detection in Water

**DOI:** 10.3390/polym15071804

**Published:** 2023-04-06

**Authors:** Nada S. Al-Kadhi, Mahmoud A. Hefnawy, Fowzia S. Alamro, Rami Adel Pashameah, Hoda A. Ahmed, Shymaa S. Medany

**Affiliations:** 1 Department of Chemistry, College of Science, Princess Nourah bint Abdulrahman University, P.O. Box 84428, Riyadh 11671, Saudi Arabia; 2 Chemistry Department, Faculty of Science, Cairo University, Giza 12613, Egypt; 3 Department of Chemistry, Faculty of Applied Science, Umm Al-Qura University, Makkah 24230, Saudi Arabia

**Keywords:** nitrite sensing, electrochemical sensor, conducting polymer, NiO nano-flowers

## Abstract

A modified electrode with conducting polymer (Polyaniline) and NiO nanoflowers was prepared to detect nitrite ions in drinking water. A simple method was used to prepare the NiO nanoflower (NiOnF). Several techniques characterized the as-prepared NiOnF to determine the chemical structure and surface morphology of the NiO, such as XRD, XPS, FT-IR, and TGA. The activity of the electrode toward nitrite sensing was investigated over a wide range of pH (i.e., 2 to 10). The amperometry method was used to determine the linear detection range and limit. Accordingly, the modified electrode GC/PANI/NiOnf showed a linear range of detection at 0.1–1 µM and 1–500 µM. At the same time, the limit of detection (LOD) was 9.7 and 64 nM for low and high concentrations, respectively. Furthermore, the kinetic characteristics of nitrite, such as diffusion and transport coefficients, were investigated in various media. Moreover, the charge transfer resistance was utilized for nitrite electrooxidation in different pH values by the electrochemical impedance technique (EIS). The anti-interfering criteria of the modified surfaces were utilized in the existence of many interfering cations in water (e.g., K^+^, Na^+^, Cu^2+^, Zn^2+^, Ba^2+^, Ca^2+^, Cr^2+^, Cd^2+^, Pd^2+^). A real sample of the Nile River was spiked with nitrite to study the activity of the electrode in a real case sample (response time ~4 s). The interaction between nitrite ions and NiO{100} surface was studied using DFT calculations as a function of adsorption energy.

## 1. Introduction

Nitrite is considered a hazardous material that drastically affects humans and the environment. However, sodium nitrite is a powerful antioxidant widely used as a food-grade additive in the food industry [1]. Moreover, nitrite is an essential ingredient in the fertilizer industry [2]. As a result of human activities, the surface and groundwater can be contaminated by leakage of excess nitrite solution. The World Health Organization (WHO) recommended that the amount of nitrite in drinking water must be less than 3 mg L^−1^. Consequently, an increase in nitrite concentration levels causes methemoglobin to be produced and reduces red blood cell oxygen-carrying capacity [3]. Moreover, nitrite can generate carcinogenic nitroso compounds, such as N-nitrosamines in the intestine and stomach [4]. 

The detection of nitrite is regarded as an essential step for contamination monitoring. Several techniques have been employed for nitrite detection, such as colorimetric [5], high-performance liquid chromatography (HPLC) [6], and electrochemical methods [7]. 

The electrochemical methods are reported to be highly sensitive and selective techniques for nitrite detection in contaminated water. Thus, there are many advantages, such as low cost, fast response, simplicity, and reproducibility [8,9]. 

Metal oxides are extensively mentioned in literature as an electrocatalyst for detecting nitrite ions. Accordingly, transition metal oxides are the most used metals for electrode preparation in nitrite electrochemical detection. However, the disadvantage of transition metal oxides is the wide bandgap that leads to poor electron transport kinetics. Therefore, the criteria of metal oxides can indeed be improved by combination with other carbon-based materials such as polymers, CNT, and graphene [10,11]. 

Nickel oxide, a highly abundant material, has received significant consideration as an electrode for electrochemical detection because of its excellent properties, such as low cost, facile synthesis, and low toxicity. Accordingly, NiO composites are mentioned extensively for nitrite detection, including NiO/MWCNT [12], NiO@graphene oxide [13], and NiO/CNT/PEDOT [14]. 

Conducting polymers are found to have fascinating properties compared with other supporting carbon materials. At the same time, the conducting polymers exhibit high electron transfer and exceptional catalytic ability [15]. Because of its excellent electrical conductivity, relative stability, and facile doping-dedoping process, polyaniline (PANI), the highly popular conducting polymer, has been frequently documented in electrochemical detection [16,17].

Furthermore, combining a metal oxide and conducting polymer (i.e., PANI) has received much attention owing to the interactive effect of electronic structure metal oxide and PANI composite compared with pristine counterparts [18]. Consequently, metal-polymer composites were reported in the literature for the efficient nitrite detection in water, such as SnO_2_/PANI [19], Au-PANI [20], PANI@SiO_2_ [21], RGO/MnFe_2_O_4_/PANI [22].

In this work, the electrode surface was modified by layers of PANI and NiO nanoflower to enhance the efficiency of nitrite detection in water. A thin layer of conducting polymer was placed on the surface of the electrode, followed by casting a layer of NiO. The synergistic effect between PANI and NiO was studied at different pH values. The different kinetic parameters were studied, such as the diffusion coefficient and charge transfer. The charge transfer resistance was estimated using the electrochemical impedance spectroscopy technique. Moreover, the effect of interfering species in the solution was characterized to determine the electrode’s anti-interfering ability. The recovery of the electrode was studied by spiking nitrite in the Nile River water. 

## 2. Materials and Methods

### 2.1. Chemicals and Reagents

Hydrated nicked acetate (Ni(ac)_2_·4H_2_O), Sodium sulfate (Na_2_SO_4_), Ammonium hydroxide (NH_4_OH), Urea, Sodium hydroxide (NaOH), isopropyl alcohol, ethanol, K_2_HPO_4,_ KH_2_PO_4_, H_3_PO_4_, Nafion solution (5%.), Aniline, and Sulfuric acid, were used as received. All solutions were prepared with double distilled water.

### 2.2. Preparation of NiO Flowers

Nickel oxide nanoflower was prepared using the following methods. Initially, 0.3 g of nickel acetate and 0.15 g of urea were dissolved in 20 mL of ethanol by heating the solution under stirring at 35 °C for 30 min until a clear solution was achieved. The resulting solution was then transferred to a stainless-steel Teflon-lined container. The sealed system was heated in an oven at 180 °C for 12 h. The system was then naturally cooled down to room temperature, and the residue was filtered and washed multiple times with 100% ethanol. The green powder was calcinated in a muffle stove at 450 °C for 3 h. Finally, the product was washed and baked in the oven at 80 °C during the night.

### 2.3. Preparation of PANI 

PANI was prepared by conventional chemical oxidative polymerization of a double-distilled aniline. 100 mL of 1.0 N HCl was added to an ice bath, then 5 mL of aniline was added sequentially to the solution with vigorous stirring to form aniline hydrochloride. Then 20 mL of sodium persulfate (0.1 M) was added portion by portion to initiate the redox polymerization. The color of the solution was observed to change along with the progress of the polymerization step. Otherwise, the generation of heat by the exothermic polymerization process can enhance the PANI’s formation rate. After four hours of oxidative polymerization under stirring conditions, complete polymerization was achieved. Finally, the violet precipitated powder (pernigraniline)was filtered, washed with deionized water, then dried overnight in the oven.

### 2.4. Fabrication of the Electrode and Electrochemical Measurements

A glassy carbon electrode of diameter = 3 mm and 0.0707 cm^2^ surface area was used as a working electrode. First, it was polished with soft emery paper and rinsed with double distilled water and ethanol. Then the electrocatalysts were prepared by dispersing 10 mg of the catalyst powder (i.e., 50 mg NiO) in a mixture of (1 mL ethanol and 1 mL 5 wt% Nafion) using an ultrasonic bath for 1 h to prepare the cast solution. Accordingly, the conducting polymer cast solution was prepared with 10 mg of PANI in 1.0 µL of ethanol. The modified electrode was prepared as follows: GC/ PANI by casting 5 µL of PANI ink onto a glassy carbon electrode surface and kept dry in an oven at 40 °C for 2 h. GC/NiO electrode was prepared by casting 10 µL of NiO ink on the glassy carbon electrode surface, and the electrode was left to dry. Finally, GC/PANI/NiO electrode was prepared by casting a layer of conducting polymer (5 µL of PANI ink). A layer of NiO was cast over GC/PANI electrode using 10 µL of NiO ink. The reported NiO nanoflower has symbolized as NiOnF. All electrochemical measurements were achieved using Autolab PGSTAT128N. The impedance spectrum was fitted with electrochemistry software, NOVA (Version 2.1, Metrohm Autolab, Utrecht, The Netherlands). The three-electrode system was constructed with GC/PANI, GC/NiOnF, and GC/PANI/NiOnF as working electrodes, Ag/AgCl/KCl (sat.), Pt wire was used as a reference, and auxiliary electrodes, respectively. All potential values in this work were referred to a Ag/AgCl/sat. KCl reference electrode. A constant AC voltage value was adjusted during the electrochemical impedance spectroscopy measurements by applying an AC voltage amplitude of 10 mV and a frequency range from 1 × 10^4^ Hz to 0.1 Hz. 

### 2.5. Characterization

After the electrocatalyst (NiO nanoflower) preparation, the chemical structure was characterized by powder X-ray diffraction (XRD) using a Panlytical X′ Pert machine. The electrode surface characterization of metal oxide and conducting polymer samples were achieved using a Scanning electron microscope of Quanta FEG 250 (USA) instrument and an EDAX Unit (Energy Dispersive X-ray Analyses) coupled to accelerating voltage 30 kV, and magnification up to 1,000,000× and resolution for Gun). The samples were coated with gold using EMITECH k550x sputter coater for higher image resolution. XPS analysis was employed to find out the bond-type and oxidation states of electrocatalyst using K-ALPHA (Thermo Fisher Scientific, USA) with monochromatic X-ray Al K-alpha radiation 10–1350 eV, spot size 400 μm, at pressure 10^−9^ mbar with 200 eV at total spectrum pass energy and 50 eV at narrow spectrum. Thermogravimetric Analysis was utilized by SHIMADZU. The sampling was measured under N2 atmosphere at a range of temperature of 30–800 °C, where the sample was held in aluminum. IR analysis was used to characterize Polyaniline (Pani) composite in the range 400 cm^−1^ to 4000 cm^−1^ using Fourier transform infrared (FTIR) spectrometer JASCO FT/IR-4100 type A with detector TGS.

## 3. Results and Discussion

### 3.1. Characterization and Analysis

**Figure 1:**
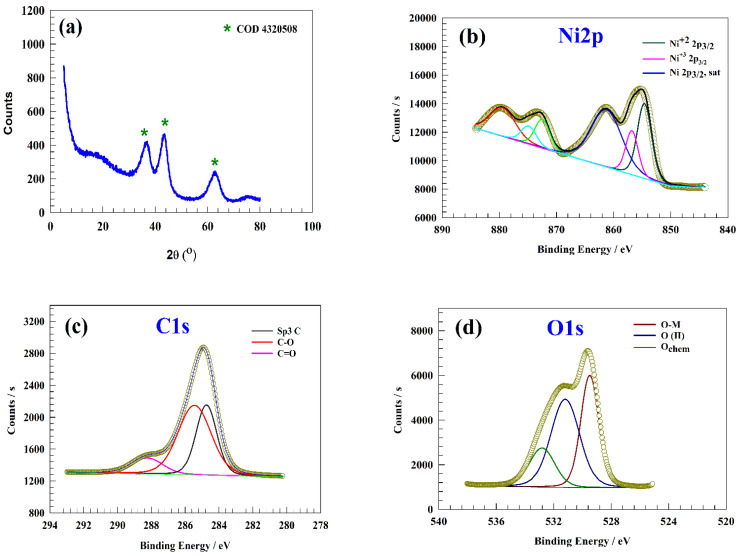
(**a**) XRD pattern of NiOnF, XPS of NiOnF spectra of (**b**) Ni2p, (**c**) C1s, and (**d**) O1s.

For confirmation of chemical structure, powder X-ray diffraction was employed to characterize the crystallographic data of NiOnF. Figure 1a shows the XRD chat of NiOnF after the preparation step. Three characteristic peaks can be observed at 2θ = 36.7, 43.3, and 63.1, corresponding to facets (111), (200), and (220) according to the COD 4320508 reference card [23]. 

X-ray photoelectron spectroscopy (XPS) characterized the prepared NiO nanoflowers’ composition. Figure 1b represents two characteristic peaks of Ni2p at 854.59 and 856.78 eV, which correspond to 2p_3/2_ Ni^2+^ and Ni^3+^ peaks, respectively [24,25,26,27]. Additionally, the corresponding satellite peak of the 2p_3/2_ was observed at a binding energy of 861.23 eV [28]. The 872.48, 874.86, and 879.45 eV peaks correspond to 2p_1/2_ and satellite of Ni2p_1/2_, respectively [29,30,31,32,33]. Figure 1c exhibits three C1s peaks with binding energies of 284.83, 285.32, and 288.58 eV. Peaks at 284.83 and 285.32 eV are associated with sp^3^ C and C-O, respectively. In addition, the presence of carbonate can be proved by the peak at a binding energy of 288.58 eV. Figure 1d represents the characteristic peak at a binding energy of 529.51 regarding the Ni-O [34,35]. The peak of 531.22 eV was attributed to the O(II) within the NiO crystal matrix [36,37,38,39]. Furthermore, the peak at 532.9 eV with low intensity corresponds to the presence of adsorbed water molecule species on the NiO nanoflower surface (O_chem_), such as H_2_O [40,41]. 

**Figure 2:**
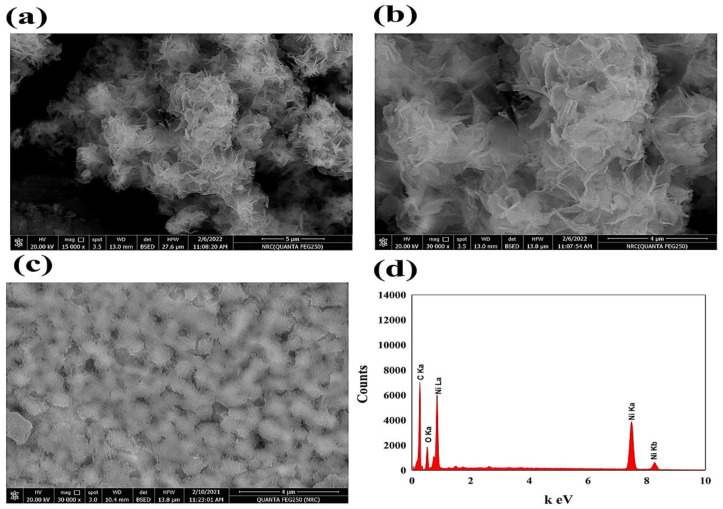
(**a**,**b**) SEM images of NiOnF at different magnifications, (**c**) PANI, (**d**) EDAX spectrum of NiOnF.

The prepared electrocatalysts’ surface morphology was considered using a scanning electron microscope (SEM). Figure 2 shows the SEM of NiO nanoflowers and PANI surfaces. In the NiOnF illustrated in Figure 2a,b, the highly porous structure of the NiOnF confirms the high activity of the modified electrode, which is connected to the high and extended surface area of the electrocatalyst. Moreover, the SEM of the PANI surface-modified layer is represented in Figure 2c. The surface’s curvatures support the NiO’s stability on the surface, where the small cavities diameter on the PANI layer was found to be in the range of 0.12~0.15 µM. 

The elemental analysis was represented In Figure 2d, as the EDAX chart reflects the presence of Ni, O, and carbon with a ratio that agrees with the presence of NiO composite. The result in the SEM section confirms the unique geometrical structure of the prepared electrocatalyst, which significantly affects the electrochemical detection of nitrite. 

**Figure 3:**
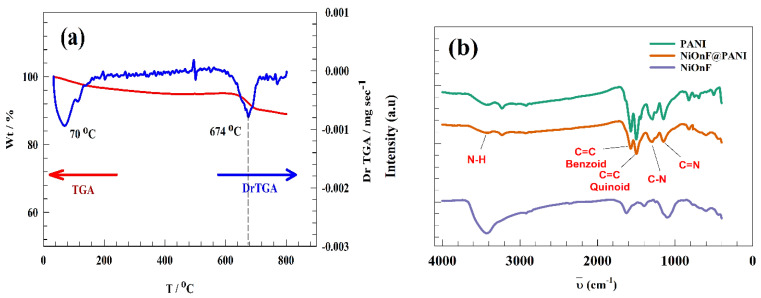
(**a**) TGA curve of the NiOnF, (**b**) FT-IR of PANI, NiOnF@PANI, and NiOnF.

Figure 3a illustrates the thermogravimetric analysis (TGA) of the as-prepared NiOnF. During the thermal treatment in the nitrogen atmosphere, significant weight loss was observed in the temperature range 610–720 °C with 6% of the starting weight loss. The differential curve in the TGA data reflects the two major transitions: the first transition at 70 °C for loss of water of crystallization. The second transition for nickel oxide sintering and removing carbonaceous materials accumulated in the preparation step. The FT-IR spectroscopy technique was employed to detect the bond stretching of the functional group within the PANI. As represented in Figure 3b, several peaks were observed at a wavenumber of 3406, 1572, 1490, 1300, and 1152 cm^−1^, corresponding to N-H stretching, Benziod ring vibration, Quinoid ring vibration, C-N, and C=N groups, respectively [42,43,44,45]. The interaction between NiOnF and PANI was observed in the IR chart. Thus, the intensity of peaks at a wavenumber of 1572, 1490, 1300, and 1152 ${\mathrm{cm}}^{-1}$ decreased. Additionally, a peak shift of the N-H band reflects the interaction between N-M. 

For NiOnF, peaks in the range of 1365 to 1032 cm^−1^ refer to the presence of some overtone. The peaks at 1610 ${\mathrm{cm}}^{-1}$ to 1620 ${\mathrm{cm}}^{-1}$ correspond to HO-H bonding. The peaks 3416 ${\mathrm{cm}}^{-1}$ correspond to the air‘s OH water and carbon dioxide bonds.

### 3.2. Study of Electrochemical Detection of Nitrite 

**Figure 4:**
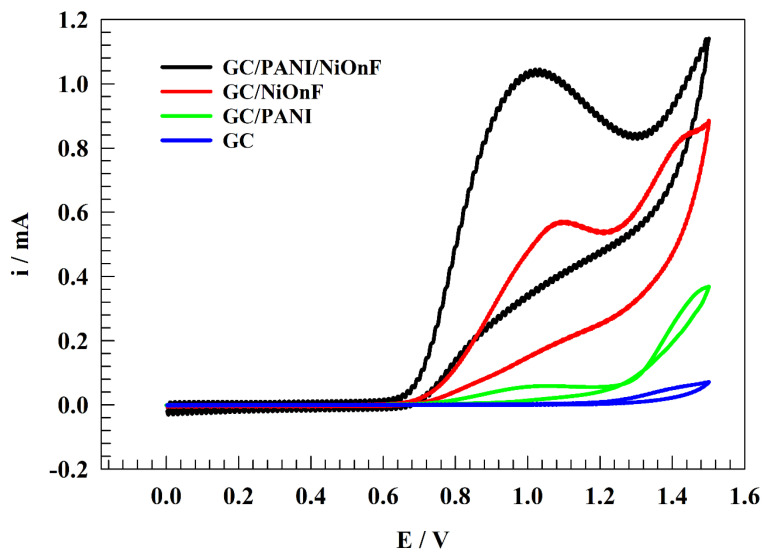
CVs of the different electrodes (GC/PANI/NiOnF, GC/NiOnF, GC/PANI and GC) in solution of 0.1 M PBS (pH = 7) and 0.5 mM nitrite at scan rate 20 mV $s^{-1}$.

The efficiency of different modified electrodes, namely GC/PANI/NiOnF, GC/NiOnF, GC/PANI, and GC, was investigated toward the detection of nitrites by the cyclic voltammetry (CV) technique in a solution of (1/10) M phosphate buffer (Neutral) holding 0.5 mM of nitrite at 20 mV$\;s^{-1}$ sweep rate (see Figure 4). Nickel-modified electrodes were firstly electrochemically treated in 0.1 M NaOH solution to generate electroactive species, namely nickel oxyhydroxide, which is responsible for the electrochemical oxidation of many species upon the nickel surfaces. The higher detection efficiency was achieved by adding a layer of PANI on the modified electrode. Accordingly, the onset potential shifted to the less positive value, which is more thermodynamically favored for electrochemical reactions. Furthermore, the maximum peak current increased, and the peak morphology became more defined because of the role of PANI in electrochemical oxidation, such as promoting the charge transfer coefficient and adsorption of nitrite ions on the solution. PANI, as carbonaceous material, improves the durability and computability between the NiOnF and glassy carbon electrodes. PANI was observed to have activity toward nitrite detection (see Figure 4), where the PANI was previously mentioned in the literature as a stand-alone electrode for nitrite detection owing to the extended functional group that can form inter-molecular interactions with the nitrite ions in the solution [46,47]. The electrochemical oxidation of nitrite upon the modified electrode was observed as a redox peak at a potential of ca. 1.0 V (vs. Ag/AgCl/sat. KCl). Thus, the forward peak corresponds to the conversion of N$O_{2}^{-}$ to N$O_{3}^{-}.$ However, the undefined backward peak and the absence of reduction peaks indicate the irreversibility of the electrochemical detection of nitrite.

The electrochemical oxidation of nitrite mainly depends on several steps, such as diffusion of nitrite ions from the bulk solution to the electrode surface followed by adsorption of ions on the electrode surfaces. The adsorption of the nitrite over the nickel oxide surface was utilized by density function theory (DFT). The computational modeling was investigated by spin-polarized calculation of the modified Revised Perdew-Burke-Ernzerhof model for the gradient density approximation [48]. The adsorption energy calculation was achieved by Cambridge Serial Total Energy Package [49]. The calculation was performed upon NiO with a crystal facet of {100}. At the same time, OTFG ultrasoft pseudo-potentials were applied on 3 × 3 supercells. The crystal was prepared by 3 layers of NiO under an energy cut-off equal to 570 eV. Otherwise, the K-points were selected as 2 × 2 × 1. The convergence parameters were optimized for the k-points grid, energy cut-offs, and layer thickness. 

The adsorption energy of the nitrite upon the NiO{100} surfaces was estimated by the following relation [50,51]:

E_ads_ = E_nitrite-NiO_ − E_nitrite_ − E_NiO_ (1)

where E_ads_: nitrite adsorption energy, E_nitrite-NiO_: energy of nitrite adsorbed on Ni{100} system, E_NiO_: energy of the clean Ni{100} surface. 

**Figure 5:**
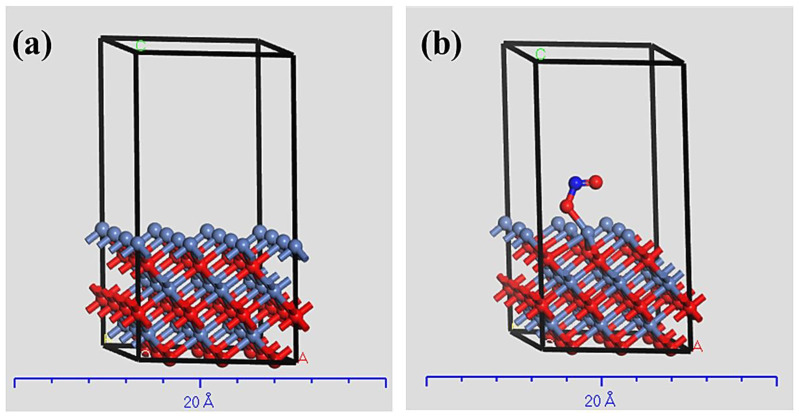
(**a**) Clean NiO{100} surface, (**b**) N$O_{2}^{-}$ adsorbed on NiO{100} surface.

Represented in Figure 5 is the crystal system of clean NiO{100} and nitrite adsorbed on NiO{100} surface. The adsorption of nitrite ions was evaluated as a function of adsorption energy. Thus, the adsorption energy of nitrite is −2.78 eV. The negative sign of the adsorption indicates the interaction between the O atom of nitrite and the nickel atoms in NiO{100} surface.

### 3.3. Effect of pH Change 

**Figure 6:**
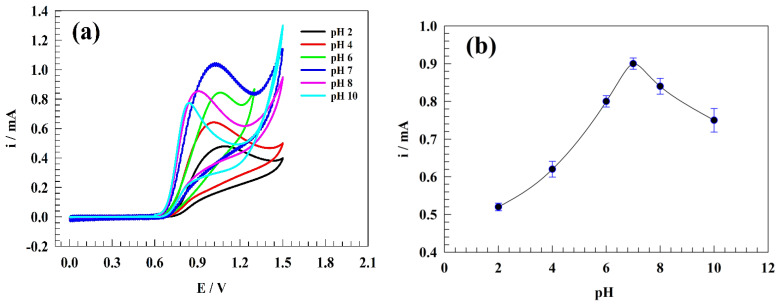
(**a**) CVs of the GC/PANI/NiOnF in 0.5 mM nitrite solution at different pH values. (**b**) The dependence of oxidation current on the pH of the solution.

The solution’s pH is an essential parameter for nitrite sensors. The presence of hydrogen or hydroxide ions in the medium could promote the modified electrode surface’s stability and efficiency. Furthermore, the diffusion of charged ions toward the electrode surface can be extensively affected by the ions that exist in the solution. Figure 6a shows the CVs of modified electrode GC/PANI/NiOnF in a solution of 0.5 mM nitrite and 0.1 M phosphate buffer at various values of pH (i.e., 2, 4, 6, 7, 8, 10) at a sweep rate of 20 mV $s^{-1}$. As the pH increases, the current of nitrite oxidation is found to be raised. The maximum oxidation current was attained at pH = 7. Moreover, the oxidation current for the basic medium was observed to be more than the acidic counterparts (see Figure 6b). The neutral and basic medium is favored for nickel-based surfaces due to the NiOOH/Ni(OH)_2_ electroactive species conversions (as reported in previous sections) [52,53,54,55]. However, the electrooxidation of nitrite was reported to be optimum in a slightly acidic, slightly basic, or neutral medium [9,56,57,58]. 

**Figure 7:**
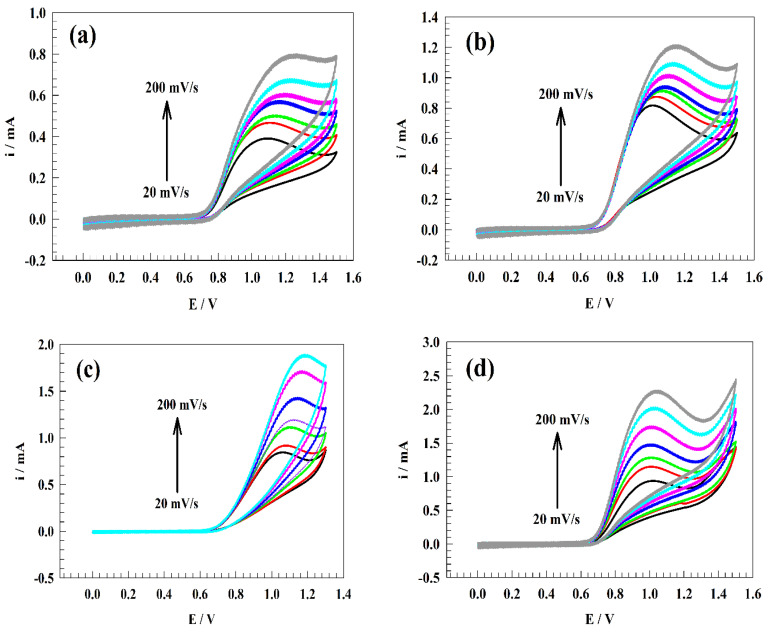

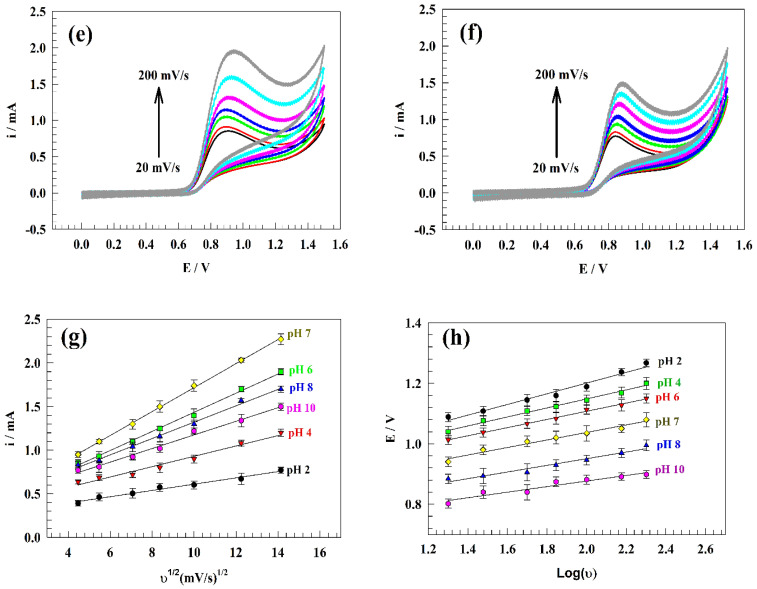
CVs of modified electrode GC/PANI/NiOnF at various sweep rate ranges (20 to 200 mV $s^{-1}$) in 0.5 mM nitrite solution and 0.1 M PBS at different pH ranges (**a**). pH 2, (**b**). pH 4, (**c**). pH 6, (**d**). pH 7, (**e**). pH 8, and (**f**). pH 10). (**g**) The relation between the square root of sweep rate and nitrite’s oxidation current. (**h**) The relation between the Log of sweep rate versus nitrite’s oxidation potential.

Since the pH of the medium significantly impacts the chemical kinetics of the nitrite oxidation, different kinetics parameters were studied as a function of pH, such as the diffusion coefficient and transfer coefficient. In Figure 7a–f, the CVs of the surface, GC/PANI/NiOnF, in 0.5 mM of Nitrite + 0.1 M PBS at a broad range of sweep rates (20–200 mV $s^{-1}$) at different pH ranges (2, 4, 6, 7, 8, and 10) are illustrated.

The diffusion coefficient of nitrite toward the electrode was estimated by different sweep rates experiments using the Randles-Sevik equation as follows [59,60,61,62]:

(2) $$i_{p}=2.6\;\times \;10^{5}\;C\;A\;n^{3/2}{\left(D\;\nu \right)}^{1/2}$$

where: i_p_: anodic current, *n*: electron numbers, A: electrode area, *D*: nitrite diffusion coefficient, C: nitrite concentration, ν: sweep rate. 

Figure 7g shows the relation between the square root of the sweep rate and oxidation current. Thus, the provided diffusion coefficients for nitrite at different pH are 1.97$\;\times \;10^{-7}$, 3.30$\;\times \;10^{-7}$, 4.27$\;\times \;10^{-7}$, 4.91$\;\times \;10^{-7}$, 3.749$\;\times \;10^{-7}$, and 2.02$\;\times \;10^{-7}$ cm^2^ $s^{-1}$ for pH 2, 4, 6, 7, 8, and 10, respectively. 

As a result of the diffusion coefficient, the highly basic solution was observed to have a lower diffusion value due to the same charge of the $NO_{2}^{-}$ and $OH^{-}$ ions. Therefore, we conclude that the presence of O$H^{-}$ ions enhance the generation of active species of NiOOH, along with decreasing the rate of nitrite diffusion by charge repulsion. 

As represented in Figure 7h, the relation between the Log of sweep rate versus the peak potentials was studied for different pH values. Thus, the linear relation was expected Laviron equation [63]:

(3) $$E_{p}=E^{{}^{\circ}}\;+\frac{\;RT\;}{\propto nF}-\frac{2.303\;RT\;}{\propto nF}\mathrm{Log}\nu$$

where: E_p_: peak potential, *R*: universal gas constant, E°: formal potential, *T*: operating temperature, *n*: involved electrons numbers, ν: sweep rate, *F*: Faraday constant. 

The observed shift in peak potential (E_p_) to a more negative value by growing the pH of the solution is matched to the assumption of the Nernst equation. 

Furthermore, the transfer coefficient (α) is considered the kinetic parameter that deduces the reaction preference towards the oxidation/reduction direction [64]. The oxidation direction is favored, whereas (α) is less than 0.5. Consequently, the transfer coefficient was estimated by the linear relation between Log (ν) vs. Ep regarding the Laviron relation. The transfer values of nitrite detection at different solution pH were estimated and reported in Table 1.

The higher value of the transfer coefficient was expected for a pH of higher current. The lowest charge transfer value is found for lower pH and the higher transfer coefficient for the basic medium, whereas the basic medium is favored for NiOOH species. 

### 3.4. Electrochemical Impedance Spectroscopy

**Figure 8:**
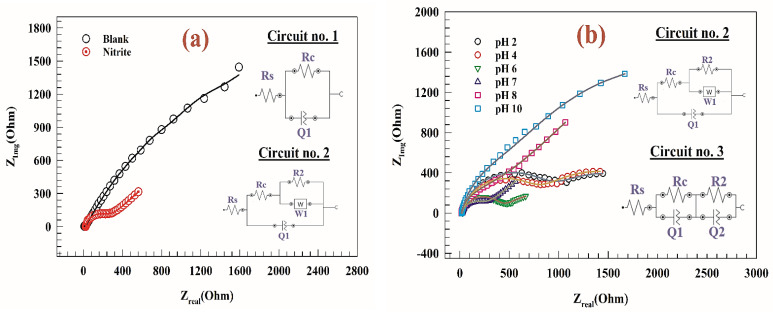
(**a**) Nyquist plots of modified electrode GC/PANI/NiOnF at constant AC potential of 1.0 V in the presence and absence of nitrite. (**b**) Nyquist plot of the modified GC/PANI/NiOnF at AC Potential of 1.0 V at different solution pH (2–10). Inset figures: the fitting circuits of the corresponding EIS result.

The electrochemical impedance spectroscopy (EIS) was studied for modified electrode GC/PANI/NiOnF in a solution of 0.1 M PBS and 0.5 mM nitrite at a constant AC potential equal to 1.0 V vs. Ag/AgCl. Figure 8a represents the Nyquist plot of a comparison between the activity of modified electrode GC/PANI/NiOnF at an AC potential of 1.0 V in the presence and the absence of the 0.5 mM nitrite. In the inset figure: circuit No.1 is attributed to the blank EIS data, and circuit No.2 corresponds to the EIS fitting data for the modified electrode in the presence of the nitrite. In circuit No.1, solution resistance (Rs) is serially connected to (R//Q), where R1 is charge transfer resistance, and Q is the constant phase element. Circuit No.2 for EIS in the presence of nitrite, including solution resistance (Rs), connected to charge transfer resistance (Rc) and constant phase element (Q). At the same time, the charge transfer resistance (Rc) is connected to the double layer resistance (R2) and Warburg diffusion element (W). At the same time, circuit No.3 consists of solution resistance (Rs) connected to two similar circuits (R//Q). Whereas the constant phase element and capacitance are almost similar parameters as follows:

(4) $$Z=\frac{\left(1/Y0\right)}{{\left(J\omega \right)}^{\alpha }}$$

The capacitance and constant phase element are equaled at Y0 = C and α = 1.

**Figure 9:**
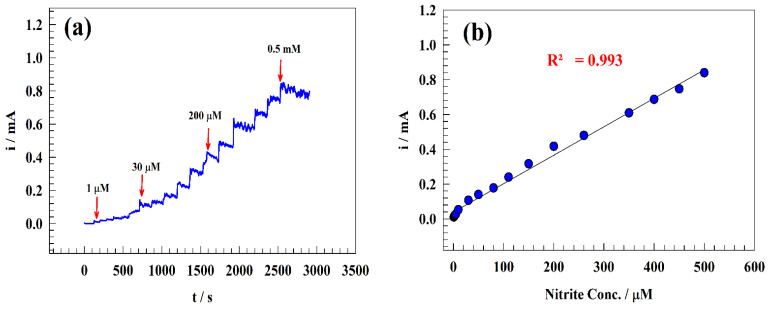

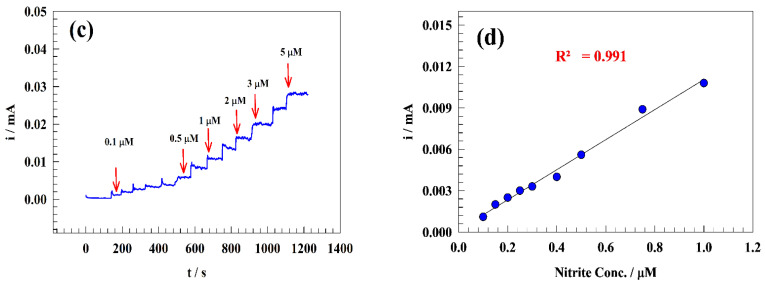
(**a**) Chronoamperogram of sequential addition of nitrite to the drinking water solution, (**b**) Typical calibration curve of nitrite detection in drinking water. (**c**) Chronoamperogram of the low concentration range for sequential addition of nitrite. (**d**) The corresponding calibration curve for low-concentration addition of nitrite ion.

The electrode was examined at different pH ranges (i.e., 2–10). Figure 8b shows Nyquist plots for modified electrodes at different pH values. According to the Nernst equation, the peak potential is likely changed by the change of solution pH. Therefore, the Nyquist plot for high pH such as at 8 and 10 showed higher resistance at the potential 1.0 V due to the CVs peak potentials being 0.91 and 0.86 V for pH 8 and 10, respectively. As seen in inset Figure 9b, the equivalent circuits for the modified GC/PANI/NiOnF electrode at different pH, circuit No.3, is favored for pH 2, 4, and 6 due to the nitrite oxidation is the demand process. The lower resistance values in the highly basic medium correspond to the high activity of the electrode in the basic medium due to the presence of NiO. Thus, nickel oxyhydroxide is favored to be generated in a basic medium. The value of different parameters of fitted EIS is listed in Table 2. It was noted that the charge transfer resistance of the modified GC/PANI/NiOnF electrode at pH = 7 is the lowest value which agrees with the CVs results in the electrochemical section. However, the reduced charge transfer resistance corresponds to quicker electron transport during the nitrite conversion [65]. Furthermore, the diameter of the semi-circuit reflects how efficient nitrite conversion whereas the more efficient process results from the minor semi-circuit diameter [15]. 

### 3.5. Effect of Nitrite Ion Concentration 

The nitrite ion detection and sensitivity calibration curve were established in a range of concentrations (from 0.1 µM to 0.5 mM). Therefore, the chronoamperometric technique was utilized to detect the limit of detection and linear dynamics range by sequentially adding nitrite to the drinking water. Figure 9a,c displays the constant potential chronoamperogram for the modified electrode GC/PANI/NiOnF in a solution of (1/10) M PBS (pH = 7) of diluted drinking water at a high concentration range of 1–500 µM (Figure 9a) and low nitrite concentration range 0.1–1 µM (Figure 9c). The relation between the nitrite concentrations versus oxidation current was illustrated in the calibration curve for the two ranges (see Figure 9b,d). 

Two linear ranges were observed for the modified electrode for 1–500 µM (Equation (5)) and 0.1–10 µM (Equation (6)) as follows:

Ip (μA) = 1.64 C_Nitrite_ (μM) + 38.47 (5)

Ip (μA) = 10.93C_Nitrite_ (μM) + 0.16 (6)

Moreover, the detection and quantization limits (knowing standard deviation s) are estimated using the equations LOD = 3 s/m, LOQ = 10 s/m, respectively.

Where: m is the calibration curve slopes. 

For GC/PANI/NiOnF designed electrodes, LOD and LOQ were obtained as 0.064 and 0.213 μM, respectively, for a range of 1–500 µM. At the same time, the LOD and LOQ were estimated to be equal to 0.0097 and 0.032 μM, respectively, for the concentration range 0.1–1 µM.

A comparison between the linear dynamic range and limit of detection between the modified GC/PANI/NiOnF electrode for determination of nitrite versus the other results reported in published literature was presented in Table 3.

### 3.6. Interference 

**Figure 10:**
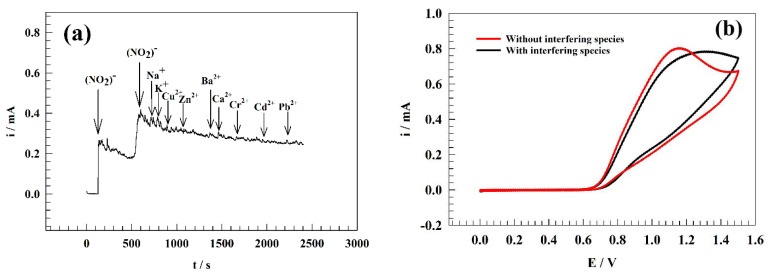
(**a**) Chronoamperogram of the modified GC/PANI/NiOnF electrode in the existence of different interfering ions. (**b**) CVs of GC/PANI/NiOnF in the presence and absence of interfering species.

The anti-interference efficiency of the GC/PANI/NiOnF surface was utilized in a solution containing a high concentration of common metal cations interfering species that extensively exist in water samples (i.e., K^+^, Na^+^, Cu^2+^, Zn^2+^, Ba^2+^, Ca^2+^, Cr^2+^, Cd^2+^, Pd^2+^). Figure 10a represents the chronoamperogram of the modified electrode after interfering species spiking in the solution. Thus, the current observed has no significant change with the addition of interfering species.

The mono-valence cations such as Na^+^ and K^+^ were observed slightly jumping in current value due to their high electrical conductance. This is in addition to the relative stability that Na and K ions can offer for nitrite ions. Other di-valence metals showed a lower change in current due to their corresponding anions, including Cl^−^, that might have interfered in this potential range. Moreover, the surface was examined by cyclic voltammetry before and after adding the previously mentioned interfering species. As represented in Figure 10b, CV of GC/PANI/NiOnF in a solution of 0.1 M PBS and 0.5 mM of nitrite in the presence and absence of the cations at a sweep rate of 20 mV $s^{-1}$. Due to some of the salt having acidic or basic preparties, the pH of the solution can shift the oxidation peak position and oxidation current value. Consequently, the medium’s pH was adjusted after adding interfering species. The oxidation current of nitrite decreased by ~5% of the initial current after adding interfering species that indicate the high anti-interfering ability of the electrode toward other metal ions in the water. 

### 3.7. Real Sample Measurement

We studied nitrite detection in distilled and drinking (tap) water. In contrast, the water sample, including brackish and surface water, contained several contaminants which affected the detection steps, such as microorganisms, organic compounds, and contaminants. 

**Figure 11:**
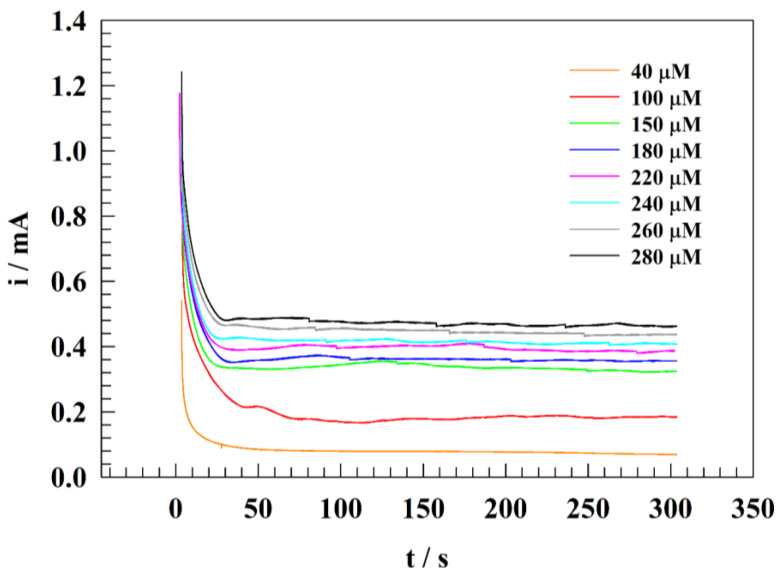
Constant potential chronoamperogram for adding different nitrite concentrations in the Nile River water at pH =7 and constant potential of 1.0 V.

The water sample was diluted with PBS, and the pH was adjusted to approximately 7. The real nitrite samples were spiked to Nile River water (conductance 465 µS) and compared to the results from the calibration curve. Figure 11 displays the chronoamperogram of wide ranges of nitrite concentrations for GC/PANI/NiOnF at a constant oxidation potential equal to 1.0 V (vs. Ag/AgCl). The oxidation current was measured by chronoamperometry of different concentrations (40 to 280 µM). The estimated results were matched with the calibration curve to find out the recovery listed in Table 4.

## 4. Conclusions

NiO nanoflower-modified PANI composite showed impressive activity toward water nitrite sensing. The addition of conducting PANI was found to enhance the detection of nitrite. In contrast, the potential of the peak and onset potential of oxidation shifted toward a more negative value which is appreciable from a thermodynamic point of view. Furthermore, the peak became more defined and higher in current. The detection of nitrite was studied at various pH values. The neutral and basic medium was observed to have a higher oxidation current because of the generation of NiOOH active species, which is favored in a basic and neutral medium. The electrode exhibits a small value of detection limit achieved at 9.7 nM and a high diffusion coefficient of 4.91$\;\times \;10^{-1}$ cm^2^ $s^{-1}$. The modified GC/PANI/NiOnF has excellent anti-interference ability in the existence of several metal ions. Moreover, the recovery of the modified electrode was investigated in the Nile River water by spiking with nitrite, which gives an acceptable value reflecting the effectiveness of the electrode (response time ~4 s). 

## Figures and Tables

**Table 1:** Representation of Diffusion coefficient, Transfer coefficient, Onset potential, Ep, and Ip for modified GC/PANI/NiOnF for different pH values.

pH	Diffusion Coefficient/D (cm^2^ s^−1^)	Transfer Coefficient/α	Onset Potential (mV)	E_p_ (mV)	I_p_ (mA)
2	1.97 × 10^−7^	0.307	0.714	1.08	0.392
4	3.30 × 10^−7^	0.401	0.691	1.04	0.636
6	4.27 × 10^−7^	0.425	0.686	1.05	0.841
7	4.91 × 10^−7^	0.596	0.682	1.04	1.023
8	3.75 × 10^−7^	0.716	0.661	0.91	0.832
10	2.02 × 10^−7^	0.813	0.654	0.86	0.771

**Table 2:** The Fitting parameters of GC/PANI/NiOnF at different pH values.

pH	Element	R_s_	R_c_	Q_1_		R_2_	Q_2_		W	
	Unit	Ω	Ω	Y_0_	*n*	Ω	Y_0_	*m*	mMho s^1/2^	χ²
2		20.04	840.76	3.35 × 10^−6^	0.868	1283.27	8.45 × 10^−5^	0.670		0.0383
4		23.51	1385.41	7.06 × 10^−6^	0.667	707.28	2.74 × 10^−6^	0.867		0.0436
6		26.11	383.99	2.09 × 10^−6^	0.853	928.50	4.58 × 10^−5^	0.864		0.0278
7		23.56	224.70	5.93 × 10^−6^	0.895	880.35			18.7 × 10^−5^	0.0231
8		14.13	356.49	9.61 × 10^−6^	0.914	601.79			7.15 × 10^−5^	0.0602
10		19.318	496.52	2.83 × 10^−5^	0.897	3527.36			2.14 × 10^−5^	0.0478

**Table 3:** Comparison between our results and other results reported in the literature for nitrite detection.

Electrode	Linear Detection Range (µM)	Limit of Detection (µM)	Method	Reference
MoO_3_/Co_3_O_4_	0.3125–4514	0.075	Amperometry	[66]
Zn-Schiff base	2–4838	0.78	Amperometry	[9]
palladium/zinc oxide/graphene oxide	3.17–1111	2.39	Amperometry	[67]
La_2_O_3_-CeO_2_	0.25–4000	0.015	Amperometry	[68]
AuNPs@MoS_2_/rGO	0.2–2600	0.038	Amperometry	[69]
PdO-RGO	10–1500	10.14	differential pulse voltammetry	[70]
ZnO/Nafion	0.3–6140	0.21	Amperometry	[71]
Fe_3_O_4_@Pt	21.1–13,000	0.109	Amperometry	[72]
Cobalt nanoflowers	100–2150	1.2	Amperometry	[73]
SiO_2_@Fe_3_O_4_	0.72–110	0.74	Amperometry	[74]
GC/PANI/NiOnF	0.1–1	0.0097	Amperometry	This work
GC/PANI/NiOnF	1–500	0.064	Amperometry	This work

**Table 4:** Real sample electrochemical detection of nitrite in the Nile River water.

Concentration of Nitrite Added (μM)	Concentration of Nitrite Found (μM)	Recovery (%)
40	42.3	105.75
100	103	103
150	160	106.4
180	194	107.8
220	216	98.2
240	231	96.25
260	265	101.9
280	272	97.1

## Data Availability

The data used for research described in this manuscript are available upon request from corresponding authors: Shymaasamir80@cu.edu.eg; shymaa@sci.cu.edu.eg; (S.S.M). maadel@sci.cu.edu.eg; maahefnawy@gmail.com. (M.A.H).
